# Effects of Chemotherapy on Hematological Parameters and CD4^+^/CD8^+^ Ratio in Cats with Mediastinal Lymphoma and Seropositive to Feline Leukemia Virus

**DOI:** 10.3390/ani12030223

**Published:** 2022-01-18

**Authors:** Tassanee Jaroensong, Juthaporn Piamwaree, Panpicha Sattasathuchana

**Affiliations:** Department of Companion Animal Clinical Sciences, Faculty of Veterinary Medicine, Kasetsart University, 50 Ngamwongwan Rd., Lat Yao, Chatuchak, Bangkok 10900, Thailand; juthaporn.pi@ku.th (J.P.); fvetpcs@ku.ac.th (P.S.)

**Keywords:** cat, CD4^+^/CD8^+^ ratio, chemotherapy, mediastinal, lymphoma

## Abstract

**Simple Summary:**

Mediastinal lymphoma is the most commonly diagnosed tumor in cats with feline leukemia virus infection (FeLV). The cyclophosphamide, vincristine, and prednisolone (COP) chemotherapeutic protocol is widely used and has achieved high complete-response (CR) rates with a long disease-free interval (DFI), and a long median survival time (MST), with low toxicity. The CD4^+^/CD8^+^ ratio is used to assess immunity in retroviral infected cats. This study was performed on 18 FeLV-infected cats with mediastinal lymphoma. The complete blood count, creatinine, alanine aminotransferase, and CD4^+^/CD8^+^ ratio were measured four times before treating with chemotherapy in the 1st, 2nd, 3rd, and 4th weeks. The white blood cell (WBC) counts, neutrophils, packed cell volume (PCV), and mean corpuscular volume (MCV) changed, while the CD4^+^/CD8^+^ ratio was not found to be significantly different. These results suggested that COP chemotherapy is safe as a treatment for FeLV-infected cats with mediastinal lymphoma.

**Abstract:**

The objective of this study was to evaluate the effect of the COP chemotherapeutic protocol on hematological parameters, CD4^+^/CD8^+^ ratio, and the mortality of 18 client-owned FeLV-infected cats with mediastinal lymphoma. The complete blood count, creatinine, alanine aminotransferase, and CD4^+^/CD8^+^ ratio were measured four times before treating with chemotherapy in the 1st, 2nd, 3rd, and 4th weeks. The white blood cell (WBC) counts at the 1st week were significantly different from the 2nd, 3rd, and 4th inductions (*p* = 0.0075, *p* = <0.0001, and *p* = 0.0271, respectively). The neutrophils at the 1st week were significantly different from the 2nd and 3rd inductions (*p* = 0.0179, and *p* < 0.0001, respectively). The packed cell volume (PCV) at the 1st week was significantly differed from the 2nd, 3rd, and 4th induction times (*p* = 0.0029, *p* = 0.0006, and *p* = 0.0029, respectively. The mean corpuscular volume (MCV) at the 1st week was significantly different from the 4th week (*p* = 0.0145). We found that chemotherapy did not cause any significant change in the CD4^+^/CD8^+^ ratio (*p*-value 0.7407). The Kaplan–Meier curves showed the median survival time (MST) for the cats with a CD4^+^/CD8 ratio of less than 1 after the 1st week of chemotherapy was 134 days. This suggested that COP chemotherapy was a safe treatment for FeLV-infected cats with mediastinal lymphoma.

## 1. Introduction

Lymphoma, one of the most common tumors in cats [1,2,3,4], is a diverse group of neoplasms emerging from lymphoreticular cells, and can be classified as nodal (mediastinal or multicentric) or extranodal (thymic, gastrointestinal, ocular, renal, cutaneous, or spinal) [5]. Approximately 70% of cats with lymphoma are infected with feline leukemia virus (FeLV) [2,6].In Bangkok, Thailand FeLV is highly prevalent at nearly 25% [7]. Mediastinal lymphoma involves the thymus, mediastinal, and sternal lymph nodes. This anatomic form is commonly found with reported levels of 20–40%, 10–50%, and 70% in the United States, England, and Japan, respectively [2]. The mediastinal form commonly occurs at a young age and is seropositive to FeLV patients [2]. Although there is no standard for the treatment of intermediate-to-high-grade mediastinal lymphoma, chemotherapy is commonly used in cats. The high-dose cyclophosphamide, vincristine, and prednisolone (COP) chemotherapeutic protocol is widely used and has achieved a high complete response (CR) rate, long disease-free interval (DFI), and long median survival time (MST), with low toxicity [1,2,8]. The MST for lymphoma without FeLV infection is in the range 105–388 days [1,9,10], but for lymphoma with FeLV infection, it is in the range 37–126.3 days [1,9,11].

Depleted T cell populations have been reported during cancer progression in humans [12]. CD4^+^ and CD8^+^ T cells are the main immune cell lymphocytes (cell-mediated immunity [CMI]). They play a major role in the tumor-response process [13,14,15]. A reduction in the CD4^+^/CD8^+^ ratio in cancer patients is considered one of the T-cells immune risk phenotypes which is related to an increase in morbidity and mortality rate in humans [16]. In cats, the CD4^+^/CD8^+^ ratio is used to assess immunity, including the pregression of feline immunodeficiency virus (FIV) and FeLV. FIV-infected cats have an inverted CD4^+^/CD8^+^ ratio that is less than 1 due to the ratio involving a selective reduction in CD4^+^ T cells, while CD8^+^ cells are relatively normal [17]. The median CD4^+^/CD8^+^ ratio determined from flow-cytometric analysis from 39 normal cats was 1.13. Unlike FIV, FeLV did not cause an inversion of the CD4^+^/CD8^+^ ratio [17,18]. A study in normal humans showed that the CD4^+^/CD8^+^ ratio in bone marrow and blood was 1 [19] and 1.5 [20], respectively. The mean blood CD4^+^/CD8^+^ ratios in bone marrow-positive and -negative follicular lymphoma patients were 1.53 and 1.57, respectively [21]. From our experience, FeLV-infected cats with mediastinal lymphoma had a poor prognosis, despite chemotherapy.

The objectives of this study were to evaluate the effect of the COP chemotherapeutic protocol on hematological parameters, the CD4^+^/CD8^+^ ratio, and mortality in FeLV positive cats with mediastinal lymphoma.

## 2. Materials and Methods

### 2.1. Ethical Approvals and Animals

This research was approved by Kasetsart University’s Institutional Animal Use and Care (ACKU 61-VET-090). Informed consent from clients was obtained in all cases. Eighteen client-owned cats with mediastinal lymphoma were enrolled in the study. Mediastinal lymphoma was confirmed using the combination test of thoracic radiography or ultrasonography and the cytological analysis of pleural effusion during May 2017 and May 2019 at the Kasetsart University Veterinary Teaching Hospital, Bangkok, Thailand. The staging was based on liver or spleen involvement after performing ultrasonography [22]. The aspiration of bone marrow, spleen, or liver was not performed for the confirmation of involvement [2,8].

All enrolled cats received COP chemotherapeutic protocol as previously described [23]. All cats received a combination of vincristine (0.5–0.75 mg/m^2^, IV) cyclophosphamide (300 mg/m^2^, PO), and prednisolone (50 mg/m^2^, PO). Additionally, all cats in this study received recombinant feline interferon omega (rFeIFN-ω) at an oral dose of 10,000 unit/day, during chemotherapy. Clinical signs were observed to obtain the clinical score (CS) at the 1st week and 4th week. CS were classified to 3 clinical groups (CG): CG1, with CS = 0 (asymptomatic); CG2, with CS = 1–5 (mild disease); CG3, with CS ≥ 6 (severe disease) [24]. Complete history taking, physical examination, thoracic radiography or ultrasonography, and rapid test kit for FeLV antigen and FIV antibody (WITNESS^®^ FeLV-FIV) were performed, and relevant data were recorded for all cats by licensed veterinarians. Cats that were negative using the test kit FeLV antigen had a whole-blood PCR analysis for FeLV provirus. Approximately 3 mL of whole blood was collected from the jugular vein, cephalic vein, or median saphenous vein and distributed to ethylenediaminetetraacetic acid (EDTA)-coated tubes and plain glass tubes for the analysis of complete blood count (CBC) and blood-smear examination, serum biochemistry panel (creatinine and alanine aminotransferase [ALT]), and measurement of CD4^+^ T-cells and CD8^+^ T-cells before performing chemotherapy in the 1st, 2nd, 3rd, and 4th weeks of the treatment. CBC was performed using an automated hematology analyzer (Abbott CELL-DYN 3700 Hematology Analyzer, Abbott, Germany). Serum biochemistry was performed using a laboratory chemical analyzer (ILab650 Automatic Biochemistry analyzer, Instrumental Laboratory, Lexington, MA, USA).

In the 4th week, before the maintenance phase, treatment responses were evaluated using chest radiography or ultrasonography and clinical signs. Cats were categorized as having a complete response (CR; regression of all measurable tumor and clinical signs), partial response (PR; reduction of >50% but <100% in size of the measurable tumor), or no response (NR; reduction of <50% or an increase in size of tumor) [2]. After the end of the study period, an assessment of mortality was conducted (alive or dead).

### 2.2. Measurement of CD4^+^/CD8^+^ Ratio

Each whole blood sample (3 mL) was immediately mixed with EDTA to prevent blood clotting. After this, 50 µL of the blood mixture was transferred to a 5 mL round-bottomed polystyrene tube (Falcon^®^, Corning, NY, USA) and mixed with monoclonal antibodies against feline CD4^+^ (10 µL; mouse anti-cat CD4^+^: FITC, Bio Rad Laboratories, Singapore) and CD8^+^ (10 µL; mouse anti-cat CD8+ alpha/beta: RPE, Bio Rad Laboratories, Singapore). After vortexing, the mixture was incubated in a dark room for 15 min. Then, 1000 µL of 1% lysis buffer (BD FACS™ Lysing Solution, San Jose, CA, USA) was added to lyse the red blood cells. The mixture was centrifuged at 4 °C and 1500 rpm for 5 min. The pellet was washed with 3000 µL of phosphate-buffered saline pH 7.4 (Sigma-Aldrich, Saint Louis, MO, USA) and centrifuged at 4 °C and 1500 rpm for 5 min. The cell pellet was reconstituted and fixed with 200 µL of 1% paraformaldehyde (Sigma-Aldrich) and stored in a dark room until analysis using the flow cytometer (BD FACSCalibur™, San Jose, CA, USA). The analysis of the lymphocyte subset (CD4^+^ and CD8^+^) used the flow cytometer at the King Chulalongkorn Memorial Hospital, Bangkok, Thailand. The ratio was considered to be decreased when it was <1.

### 2.3. Statistical Analysis

Commercially available statistic software packages (JMP Pro 10; SAS Institute, Cary, NC; PRISM v.6.0, GraphPad Software, La Jolla, CA, USA) were used for statistical analyses. A normality test was performed for all data using the Shapiro–Wilk W test. The Friedman test was used to evaluate the effect of chemotherapy on the CD4^+^/CD8^+^ ratio at four intervals (weekly) and was also used to evaluate the clinicopathological findings after four weeks for the cats with mediastinal lymphoma. Fisher’s exact test was used to determine the association between the CD4^+^/CD8^+^ ratio in the 1st week and mortality before receiving chemotherapy in cats with mediastinal lymphoma and was also used to determine the association between the CD4^+^/CD8^+^ ratio at the 4th week and clinical response to chemotherapy. MST was estimated based on Kaplan–Meier curves. Overall survival (OS) was determined from the first dose of chemotherapy to death related to mediastinal lymphoma. Unrelated deaths were censored, along with those lost to follow-up. *p*-values less than 0.05 were considered statistically significant.

## 3. Results

All 18 enrolled cats were positive for the FeLV antigen. Patient characteristics are shown in Table 1. The median age at diagnosis of lymphoma was 2.5 years (range 9 months–8 years). Out of the 18 cats, 13 were males (72.22%) and 5 were females (27.78%). All cats were domestic short-haired. The anatomical form and cytological evaluation of all cats with lymphoma in this study was mediastinal lymphoblastic lymphoma. The presenting clinical complaints were dyspnea and anorexia for all cats (100%), regurgitation in three cats (17%), and coughing in one cat (6%). All cats had pleural effusion and cranial mediastinal masses. The adverse events following chemotherapy, including anorexia, vomiting and diarrhea, were observed. Anorexia was observed in seven cats (39%). Vomiting was observed in four cats (22%). Diarrhea was not reported for any of the cats during follow-up. Out of the 18 cats, 7 cats had CG2, and 11 cats had CG3 at the 1st week. Thirteen cats had CG2, and 5 cats had CG3 at the 4th week. All cats also had received recombinant feline interferon-omega (IFN-ω; Virbagen^®^ 10,000-unit, PO).

**Figure 1:**
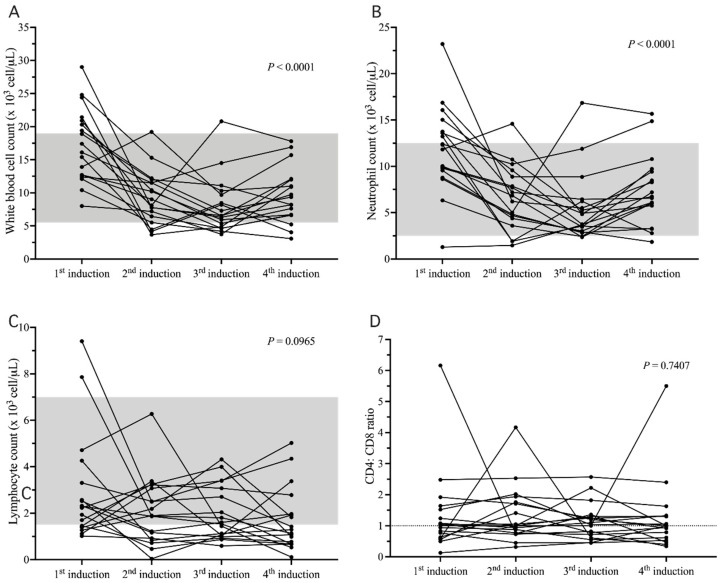
Changes in leukogram and CD4^+^/CD8^+^ ratio at 4 different times. Change in white blood cell count (**A**), where the 1st week was significantly different from the 2nd, 3rd, and 4th inductions (*p* = 0.0075, *p* = <0.0001, and *p* = 0.0271, respectively); Change in neutrophil count (**B**), where the 1st induction was significantly different from the 2nd and 3rd inductions (*p* = 0.0179, and *p* < 0.0001, respectively); Change in lymphocyte count (**C**), with no significant differences among the 4 times (*p =* 0.0965); Change in CD4^+^/CD8^+^ ratio (**D**) among 4 times, with no significant differences (*p* = 0.7407). The shaded part is the normal reference range.

After the CBC measurement (Supplementary Tables S1 and S2), values for the cats for median white blood cell count (WBC) in the 1st, 2nd, 3rd, and 4th weeks (1st induction, 2nd induction, 3rd induction, and 4th induction, respectively) were 16.75 × 10^3^ cell/µL (range 8–29 × 10^3^ cell/µL), 8.55 × 10^3^ cell/µL (range 3.67–19.2 × 10^3^ cell/µL), 6.55 × 10^3^ cell/µL (range 3.73–20.8 × 10^3^ cell/µL), and 8.79 × 10^3^ cell/µL (range 3.08–17.8 × 10^3^ cell/µL), respectively. The results showed that there was a significant difference among the four times for WBC (*p* < 0.0001; Figure 1A) and that the 1st week was significantly different from the 2nd, 3rd, and 4th weeks (*p* = 0.0075, *p* <0.0001, and *p* = 0.0271, respectively).

The median neutrophil counts in the 1st, 2nd, 3rd, and 4th weeks were 12.07 × 10^3^ cell/µL (range 1.29–23.2 × 10^3^ cell/µL), 6.53 × 10^3^ cell/µL (range 1.47–14.59 × 10^3^ cell/µL), 4.3 × 10^3^ cell/µL (range 2.36–16.85 × 10^3^ cell/µL), and 6.65 × 10^3^ cell/µL (range 1.85–15.66 × 10^3^ cell/µL), respectively. There was a significant difference among the 4 times for the neutrophil count (*p* < 0.0001; Figure 1B) and the 1st induction was significantly different from the 2nd and 3rd inductions (*p* = 0.0179, and *p* < 0.0001, respectively).

The median lymphocyte counts in the 1st, 2nd, 3rd, and 4th weeks were 2.27 × 10^3^ cell/µL (range 1.02–9.41 × 10^3^ cell/µL), 1.88 × 10^3^ cell/µL (range 0.04–6.27 × 10^3^ cell/µL), 1.57 × 10^3^ cell/µL (range 0.59–4.31 × 10^3^ cell/µL), and 1.21 × 10^3^ cell/µL (range 0.11–5.02 × 10^3^ cell/µL), respectively. There was no significant difference for the lymphocyte count between the four weeks (*p = 0.0965*; Figure 1C).

**Figure 2:**
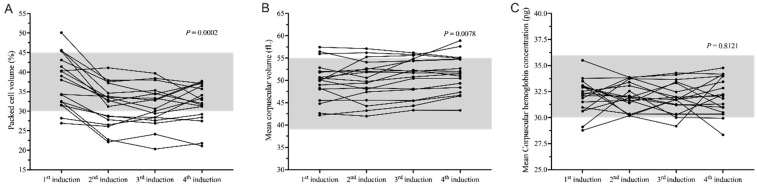
Changes in hemogram at 4 times. Change in packed cell volume (**A**), where the packed cell volume at the 1st induction time was significantly different from the 2nd, 3rd, and 4th induction times (*p* = 0.0029, *p* = 0.0006, and *p* = 0.0029, respectively); Change in mean corpuscular volume (**B**), where the 1st induction was significantly different from the 4th induction point (*p* = 0.0145); Change in mean corpuscular hemoglobin concentration (**C**), with no significant differences (*p* = 0.8121). The shaded part is the normal reference range.

The median packed-cell volumes (PCVs) in the 1st, 2nd, 3rd, and 4th inductions were 38.5% (range 26.9–50.1%), 32.65% (range 22.1–41.1%), 31.7% (range 20.3–39.7%), and 32.45% (range 21.1–37.7%), respectively. There was a significant difference among the four times of measurement for the PCV (*p* = 0.0002; Figure 2A) and the 1st induction was significantly different from the 2nd, 3rd, and 4th inductions (*p* = 0.0029, *p* = 0.0006, and *p* = 0.0029, respectively).

The median mean corpuscular volumes (MCVs) in the 1st, 2nd, 3rd, and 4th weeks were 50.11 fL (range 42.2–57.45 fL), 50.17 fL (range 41.99–57.11 fL), 51.87 fL (range 43.31–56.18 fL), and 51.58 fL (range 43.29–58.92 fL), respectively. There was a significant difference in MCV (*p* = 0.0078) (Figure 2B), and the 1st week was significantly different from the 4th week (*p* = 0.0145).

The median mean corpuscular hemoglobin concentrations (MCHCs) in the 1st, 2nd, 3rd, and 4th weeks were 32.28 pg (range 28.77–35.5 pg), 31.95 pg (range 30.17–33.87 pg), 31.74 pg (range 29.17–34.28 pg), and 32.15 pg (range 28.34–34.77 pg), respectively. However, there was no significant difference in MCHC (*p* = 0.8121; Figure 2C).

After the CD4^+^/CD8^+^ ratio measurement, the median CD4^+^/CD8^+^ ratios in the 1st, 2nd, 3rd, and 4th weeks were 1.02 (range 0.13–6.16), 1 (range 0.32–4.17), 1.07 (range 0.45–2.57), and 1 (range 0.34–5.5), respectively. There was no significant difference among the 4 times for the CD4^+^/CD8^+^ (*p* = 0.7407; Figure 1D).

The treatment response was evaluated before the 4th induction time point using thoracic radiography or ultrasonography, with 15 (83.33%) cats having a complete response and three (16.67%) cats having no response. However, there was no significant association between the CD4^+^/CD8^+^ ratio at the 4th induction and the treatment response (*p* = 0.3778). At the end of the study, 5 (27.78%) out of the 18 cats had died and 13 (72.22%) cats were still alive. There was no significant association between the CD4^+^/CD8^+^ ratio at the 1st induction and mortality (*p* = 1.000).

**Figure 3:**
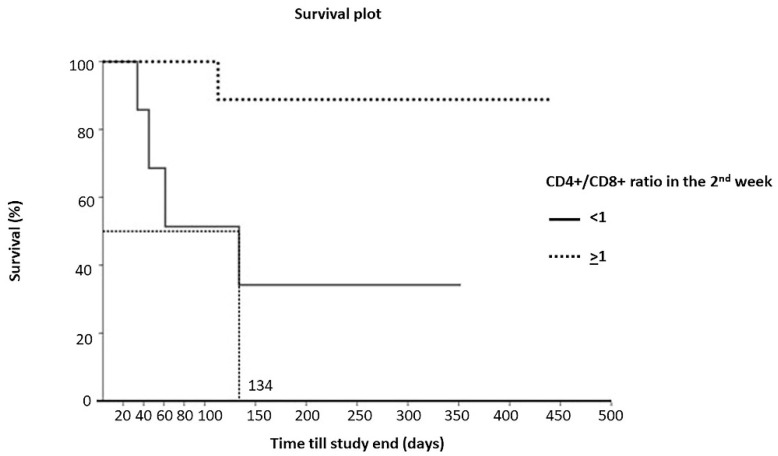
Kaplan–Meier curve for survival of cats with CD4^+^/CD8^+^ ratio of less than 1 (*n =* 7; median 134 days) or greater than or equal to 1 (*n =* 11; median not reached) after the 1st week of chemotherapy. Cox-Mantel log-rank was significantly different (*p* = 0.0173).

There were five dead cats and 13 living asymptomatic cats after treatment for 52 weeks. There were four dead cats for cats with a CD4^+^/CD8^+^ ratio after the 1st week of chemotherapy of less than 1 (*n* = 7). There was one dead cat for cats with a CD4^+^/CD8^+^ ratio after the 1st week of chemotherapy that was greater than or equal to 1 (*n =* 11). The Kaplan–Meier curve showed that the MST for the cats with a CD4^+^/CD8^+^ ratio after the 1st week of chemotherapy of less than 1 was 134 days (Figure 3). The Cox-Mantel log-rank significantly differed (*p* = 0.0173).

## 4. Discussion

Despite the large number of feline lymphoma studies, there is a paucity of information regarding cats with a high prevalence of FeLV infection [25]. In this study, we evaluated the effects of COP chemotherapy on CBC, blood chemistry, and the CD4^+^/CD8^+^ ratio in 18 cats with progressive FeLV infection. Feline mediastinal lymphoma occurs most frequently in young male cats with FeLV infection [2,23,26,27]. The present study was conducted in Bangkok, Thailand where FeLV antigenemia prevalence was estimated to be around 25% [7] with most of the sampled cats being from the streets or temples and bitten by FeLV-carrier cats before adoption by the owner. In both lymphomas with and without FeLV infection, the treatment of choice is chemotherapy [1,9]. However, FeLV infection is considered to be a poor prognostic factor for lymphoma with an MST of 37–126.3 days [1,9,11]. As in another study with a high number of cats with persistent viremia [8], all cats (18/18) in the present study tested positive for FeLV antigenemia. The median age of the cats in the present study was 2.5 years, which was consistent with other studies involving cats with mediastinal lymphoma [2,23,27]. One study suggested that a genetic factor in Siamese cats may play an important role in the tumorigenesis of mediastinal lymphoma [2]. However, as all cats in the present study were only domestic short-haired, it was difficult to conclusively identify the role of any genetic factor.

The COP chemotherapeutic protocol, which is popular and commonly used to treat lymphoma in cats [1,2,8], has achieved high CR rates, long DFIs and MSTs, and low toxicity [8]. The CR rate in other studies for cats receiving a COP protocol was 92% for Cotter et al., 81.8% for Teske et al., and 61.5% for Frabizio et al. The CR rate during the induction phase of chemotherapy in the current study was 100%.

Chemotherapy is usually well tolerated by cats and is mainly based on two phases, namely, induction and maintenance. However, the toxicity information associated with COP chemotherapy during the induction phase, based on the CBC, blood chemistry and immunological parameters (CD4^+^/CD8^+^ ratio) in FeLV-infected cats is scarce. The evaluation of toxicity based on CHOP (cyclophosphamide, hydroxydaunorubicin [doxorubicin], vincristine [Oncovin] and prednisolone) [25] and LOPH (lomustine, vincristine [Oncovin], prednisolone and hydroxydaunorubicin [doxorubicin]) [8]-based protocols in cats showed neutropenia in 46% and 76.2% of patients, respectively. In the present study, the CBC values for 4 of the 18 cats (22%) showed neutropenia. The neutrophil counts of the cats prior to receiving induction chemotherapy in the 1st week were significantly different from the 2nd and 3rd weeks (*p* = 0.0179, and *p* ≤ 0.0001, respectively). This may have been due to the adverse effect of chemotherapy. The chemotherapeutic agents associated with neutropenia in the present study may have been resultant of the vincristine and cyclophosphamide. Vincristine, a cell cycle-specific drug, can interfere with microtubule formation and mitosis, causing the lowest neutrophil counts within 7–9 days [8]. However, vincristine is associated with mild toxicity. Cyclophosphamide is a nitrogen mustard agent that is commonly combined in multiagent chemotherapeutic protocols for dogs and cats [28]. On the other hand, FeLV can infect hematopoietic stem cells in the bone marrow causing neutropenia and secondary infection infected cats [29]. The PCV and MCV values were significantly different, due to the cyclophosphamide and vincristine suppressing the bone marrow that would likely cause anemia. Furthermore, FeLV infection has been reported to be the cause of an increase in the MCV [30] from skipping mitosis in the S-phase cell cycle of erythropoiesis. Anemia is a major complication that occurs in FeLV-infected cats [29].

The ratios of CD4^+^/CD8^+^ at all 4 times during the induction phase with COP chemotherapy were not significantly different. In this research, FeIFN-ω may have played a role in modulating the host’s immune system, resulting in an unchanged ratio of CD4^+^/CD8^+^. According to another study, FeIFN-ω can improve clinical signs and hematologic parameters in naturally retroviral infected cats, but it did not change hypergammaglobulinemia, the pro-viral load and anemia, suggesting an overall effect on the innate immune reaction, rather than on acquired immunity [31]. T-helper 1 and T-helper 2 cell responses did not significantly change in either the subcutaneous or oral protocol, which supported that FeIFN-ω did not affect the acquired immunity [32]. FeLV or FIV infected cats treated with recombinant human interferon-α improved in terms of most of the parameters analyzed (clinical status, anemia, white cell counts and CD4^+^/CD8^+^ ratio) in another study [24], similar to that described previously in FeIFN-ω. FeIFN-ω is licensed as a veterinary medicine in Australia, European countries, Japan, and Thailand. There was a significant difference in the clinical signs [33] and survival time between FeLV cats subcutaneously treated three times on five consecutive days, with a high dose at 1 MU/kg q24h, and untreated FeLV cats in the nine months of the follow-up period [34]. The CD4^+^/CD8^+^ ratio of renal cell carcinoma patients treated with vinblastine in combination with human IFN-α was higher compared to those that had been treated with vinblastine alone [35]. However, to date, no study has been published on the oral use of rFeIFN-ω in FeLV-infected cats with feline lymphoma.

We observed that the COP chemotherapy in this study was well tolerated by the cats during the induction phase. More studies should be conducted with a longer observation period to evaluate the relationships among the CD4^+^/CD8^+^ ratio, tolerability, and survival time. This study was limited by the consideration of only one breed of cat, namely, the domestic short hair, and a relatively small number of specimens (*n =* 18). There was also a limit to the duration of the study. The availability of information on the CD4^+^/CD8^+^ ratio in cats with mediastinal lymphoma that have been treated using chemotherapy is inadequate due to limited studies. Therefore, further studies should be conducted to provide more data that may help to direct further treatment. Another limitation is that the staging of bone marrow, spleen, or liver involvement has not been performed. This is because all of the enrolled cats had suffered dyspnea due to mediastinum mass compression. The diagnosis and treatment must therefore be performed immediately. In order to avoid the bone marrow, spleen, or liver involvement, we enrolled the cats with single node (mediastinum) involvement.

## 5. Conclusions

The CD4^+^/CD8^+^ ratio during the induction phase of COP chemotherapy in cats with mediastinal lymphoma was not different from pretreatment, though it can cause changes in the WBC, neutrophils, PCV, and MCV. These results suggested that COP chemotherapy is safe to apply for FeLV-infected cats with mediastinal lymphoma.

## Figures and Tables

**Table 1:** Patient characteristic of 18 cats with mediastinal lymphoma.

Parameters	
Median age (years)	2.5
Gender	
Male	72.22% (*n =* 13)
Female	27.78% (*n =* 5)
Breed	
Domestic short hair	100% (*n =* 18)
Viral status	
FeLV antigen positive	100% (*n =* 18)
Tumor anatomic location	
Mediastinum	100% (*n =* 18)
Cytological evaluation	
Lymphoblastic lymphoma	100% (*n =* 18)
Clinical complaint	
Dyspnea	100% (*n =* 18)
Anorexia	100% (*n =* 18)
Regurgitation	17% (*n =* 3)
Coughing	6% (*n =* 1)

## Data Availability

The data in this study are available in the article.

## Supplementary Materials

The following supporting information can be downloaded at: https://www.mdpi.com/article/10.3390/ani12030223/s1, Table S1: Individual leukogram of the 18 mediastinal lymphoma cats in the study; Table S2: Individual hemogram of the 18 mediastinal lymphoma cats in the study.
