# Supplementary material for: Effects of Chemotherapy on Hematological Parameters and CD4+/CD8+ Ratio in Cats with Mediastinal Lymphoma and Seropositive to Feline Leukemia Virus
Source: Animals (Basel). 2022 Jan 18;12(3):223. doi: 10.3390/ani12030223 (PMC8833558; doi:10.3390/ani12030223)
Supplement: Supplementary file 1 [file animals-12-00223-s001.zip › animals-1542597-supplementary.pdf]

Table S1. Individual leukogram of the 18 mediastinal lymphoma cats in the study. ID, identification of each cat at 4 times. WBC, white blood cell count (reference range 5.5-19.0 x 10^3 cells/μL). Neu, segmented neutrophil count (reference range 2.5-12.5 x10^3 cells/μL). Lym, lymphocyte count (reference range 1.5-7.0 x10^3 cells/μL).

ID	WBCx10^3				Neu x10^3				Lym x10^3				CD4+/CD8+ ratio				Survival time (days)
	1st week	2nd week	3rd week	4th week	1st week	2nd week	3rd week	4th week	1st week	2nd week	3rd week	4th week	1st week	2nd week	3rd week	4th week	
**1**	24.8	15.3	10.3	11	16.86	8.87	8.86	10.78	4.71	6.27	1.44	0.11	0.13	0.32	0.46	1	34
**2**	16.1	11.5	14.5	16.9	12.40	10.24	11.89	14.87	1.93	0.46	0.87	0.68	0.5	1	1	5.5	alive
**3**	12.7	9.01	4.63	6.67	10.03	6.85	2.36	6.00	1.27	1.89	2.04	0.67	1.24	1	2.22	1	alive
**4**	21.42	3.67	4.86	12	1.29	1.47	3.69	9.72	2.57	0.04	1.07	1.92	0.56	1.76	1.2	1	alive
**5**	18.9	12	11.1	8.21	16.07	7.20	6.44	6.57	1.70	3.24	4.00	0.99	0.83	0.73	0.82	0.34	alive
**6**	20.9	7.79	6.31	8.2	8.78	4.83	2.84	3.28	9.41	2.49	3.41	4.35	1.08	0.94	1.1	1.33	alive
**7**	24.41	4.16	8.2	4.04	13.23	1.91	6.23	2.79	7.86	1.87	1.80	1.13	6.16	0.75	1.36	0.47	61
**8**	12.4	11.5	6.54	12.1	9.80	7.71	3.47	8.47	2.23	3.22	3.07	2.78	1	1	1.3	1.3	alive
**9**	19.4	12.2	7.3	9.36	13.58	10.74	5.26	6.74	2.52	1.22	1.61	1.97	0.59	4.17	0.61	0.4	alive
**10**	8	7.19	3.73	10.9	6.32	3.60	2.42	7.19	1.44	3.38	1.04	3.38	1.06	0.77	0.72	0.79	45
**11**	13.9	19.2	9.69	15.7	11.82	14.59	4.85	9.42	1.11	3.07	3.39	5.02	1.52	2.02	1.03	1.06	113
**12**	17.4	8.09	20.8	17.8	13.75	5.02	16.85	15.66	3.31	2.51	2.70	1.42	2.48	2.53	2.57	2.4	alive
**13**	12.1	6.44	4.64	6.61	9.56	4.38	3.02	6.02	2.30	1.87	1.53	0.53	0.94	0.88	0.55	0.62	134
**14**	10.4	5.51	4.19	3.08	8.63	4.68	2.93	1.85	1.46	0.72	0.92	0.77	1.04	1.05	1.25	0.92	alive
**15**	29	7.76	6.55	9.74	23.20	6.21	5.50	8.28	2.32	1.16	0.98	1.07	1.92	1.71	1.24	1.32	alive
**16**	20.3	10.4	5.35	7.62	15.02	9.57	3.75	6.10	4.26	0.83	1.12	1.30	1.64	1.94	1.82	1.63	alive
**17**	15.4	4.46	8.46	5.22	12.32	1.92	3.55	3.24	1.39	2.19	4.31	1.83	0.63	1.41	0.78	0.94	alive
**18**	12.7	10.2	5.93	6.69	9.91	7.85	4.92	5.75	1.02	0.92	0.59	0.67	0.77	0.45	0.45	0.53	alive

Table S2. Individual hemogram of the 18 mediastinal lymphoma cats in the study. ID, identification of each cat at 4 times. PCV, packed cell volume (reference range 30-45%), MCV, mean corpuscular volume (reference range 39-55 fl) and MCHC, mean corpuscular hemoglobin concentration (reference range 30-36 gm%).

ID	PCV				MCV				MCHC			
	1st week	2nd week	3rd week	4th week	1st week	2nd week	3rd week	4th week	1st week	2nd week	3rd week	4th week
**1**	40.3	41.1	39.7	32.9	42.2	43.22	44.31	46.53	33.5	30.17	31.74	32.22
**2**	31.6	22.1	24.1	21.1	57.45	57.11	56.18	55.09	28.77	30.18	29.17	33.98
**3**	45.4	37.9	38	35.7	50.67	51.85	51.7	51.66	31.5	31.66	30.26	32.21
**4**	31.5	28.8	27.6	29.2	45.5	45.57	45.47	47.33	32.1	32.08	31.27	30.99
**5**	28.2	26.5	28.4	27.5	50.09	48.01	50.44	50.46	32.2	33.4	31.69	34.15
**6**	34.3	33.8	32.8	37.4	44.78	47.41	47.95	49.34	32.36	31.95	31.71	28.34
**7**	34.2	27.8	26.9	28.4	49.9	55.27	55.58	57.61	33	31.62	31.97	30.49
**8**	26.9	26.1	30	33.3	48.04	48.33	48.08	48.4	30.97	30.34	30.33	30.33
**9**	43.1	37.5	38.4	37.1	48.98	49.41	52.32	50.96	35.5	33.87	34.11	34.77
**10**	50.1	37.2	34.4	37.1	51.81	52.47	54.43	54.8	32.53	33.06	31.98	32.08
**11**	40.2	33.5	33.3	31.3	48.26	44.25	45.43	46.65	29.1	32.54	30	29.94
**12**	39	32.8	29.4	31.2	50.52	49.77	50.78	51.49	33.08	31.4	33.44	31.83
**13**	45.6	34.6	35.3	31.6	42.62	41.99	43.31	43.29	33.77	33.82	34.28	34.18
**14**	32.5	22.7	20.3	21.8	52.85	50.56	54.86	58.92	30.62	33.74	33.65	33.99
**15**	45.4	31.2	33	37.7	56.47	54.07	54.37	54.72	30.62	31.89	31.21	31.3
**16**	32.4	28.6	28.4	34.1	55.96	56.19	55.8	54.91	33.02	30.28	31.73	32.84
**17**	41.4	32.5	34.5	32	52.01	52	52.19	52.03	31.88	33.85	32.46	33.44
**18**	38	33.5	30.6	36.5	50.13	52.67	52.04	52.67	32.89	31.94	33.33	30.96
